# The scientific impact of microbial cell factories

**DOI:** 10.1186/1475-2859-7-33

**Published:** 2008-12-01

**Authors:** Maurilio De Felice, Diethard Mattanovich, Maria Papagianni, Grzegorz Wegrzyn, Antonio Villaverde

**Affiliations:** 1 Department of Structural and Functional Biology, University of Naples Federico II, via Cinthia, 80126 Naples, Italy; 2 University of Natural Resources and Applied Life Sciences Vienna, Department of Biotechnology, Vienna, Austria; 3 Department of Hygiene and Technology of Food of Animal Origin, School of Veterinary Medicine, Aristotle University of Thessaloniki, Thessaloniki 54006, Greece; 4 Department of Molecular Biology, University of Gdansk, Gdansk, Poland; 5 Institute for Biotechnology and Biomedicine, Autonomous University of Barcelona, Barcelona, Spain; 6 Department of Genetics and Microbiology, Autonomous University of Barcelona, Barcelona, Spain; 7 CIBER de Bioingeniería, Biomateriales y Nanomedicina (CIBER-BBN), Bellaterra, 08193 Barcelona, Spain

## Editorial

Microbial Cell Factories was launched in 2002 under an Open Access policy, to cover a gap in the current offer of the scientific literature in Biotechnology and Applied Microbiology areas. The microbial cell factory concept, although present as a side topic within the scope of many journals in the field, deserves a specific attention as a particular, well defined issue in the microbial production and transformation of biotechnologically relevant substances. Intriguingly, the Cell Factory concept stresses the relevance of host cell genetics and metabolism in the context of the production process, and focus on the physiological aspects of the productive event. Since 2002, the journal has published more than 170 relevant manuscripts in form of Research articles, Technical notes, Reviews and Commentaries, highlighting the role of the hosting cell from both biological and technological sides. The diversity of microbial cell types being incorporated as cell factories (namely bacteria, archae, yeast and filamentous fungi), the methodological adaptation of productive processes (through new genetic engineering tools, microreactors, metagenomic approaches etc) and the diversity of fields in which cell factories become critical (structural biology, food microbiology, natural products, biominery, nanotechnology and biosensing among others), has dramatically expanded the scope covered by Microbial Cell Factories.

The journal has published excellent contributions in those areas, many of them highly cited, and it has been extremely well received by the scientific community becoming now a reference in the current microbial biotechnology literature. Thomson Reuters (ISI) has just released the first official impact factor for Microbial Cell Factories, an impressive 3.36 (for 2007), placing the journal in position 31 (out of 138 listed journals) of the Biotechnology and Applied Microbiology subject. The readers should note that in June's edition of the Journal Citation Reports (JCR) the impact factor of Microbial Cell Factories was erroneously reflected due to a failure in the system aggregating citations. The fault has been corrected in November's edition (on November 20), so the readers and potential authors should now update their records.

The journal has published relevant contributions in specific fields, some of them offering new scientific concepts or summarizing the current state-of-the-art in key methodologies and technical approaches. Regarding protein production, a special attention has been paid to recombinant protein folding and misfolding, especially in conventional hosts such as *E. coli*. In this regard, the nature, formation and physiological processing of inclusion bodies [1,2], in vitro protein refolding [3], the mechanics of bacterial quality control system [4] and the general conformational stress responses under a host comparative overview [5] have been discussed. Also, the mechanics of in vivo protein disaggregation has been extensively revised [6] and the scientific and technical implications of protein folding evaluated, conceptually [7,8] and methodologically [9-11].

Also, several authors have described the successful production of antibodies and other proteins of biotechnological interest in *Bacillus megaterium *and related species [12,13], while Zweers and coauthors have recently reviewed the use and properties of *B. subtilis *and other species as cell factory for protein production of complex proteins [14], stressing the value of this gram-positive genera as cell factory. Other hosts such as fungi [15,16], lactobacilli and lactococci [17-19] and yeasts [20] have been also revised through different examples and under diverse perspectives. Finally, novel hosts such as cold-adapted bacteria [21] or hyperthermoacidophilic archae [22] and their implementation for protein production have been evaluated. From the methodological point of view, purification, analysis of protein aggregation [23,24] and other aspects of protein production and purification have been considered [25,26], while the novelty and biotechnological interests of novel products such as the spider silk proteins [27] have been stressed.

Since the launch, Microbial Cell Factories has focussed strongly on metabolic engineering. Following the process chain, substrate utilization, and the availability of new substrates appears as the first essential topics. Improved utilization of already established substrates like glucose (reviewed in [28]) or sucrose [29] was highlighted. Utilization of the lignocellulose monomer xylose, was a topic of great interest over the last years [30-33]. Following the process chain, metabolic engineering towards the production of bioorganic molecules has been highlighted towards organic acids [34], amino acids [35], secondary metabolites [36,37] and biopolymers [38].

To establish engineered production strains, screening and analysis tools need to be applied. Mattanovich and Borth [39] reviewed single cell sorting applied to biotechnology. Analysis of transcript regulation by DNA microarrays and alternative techniques were applied to protein producing microorganisms [40], as well as amino acid [41] and antibiotics production [42]. Applications and pitfalls of transcriptomics was also reviewed [43]. Proteomics – the differential abundance of cellular proteins in different conditions – was reviewed comprehensively by Graham et al. [44]. While metabolomics methods have been established in the last decade, their application to microbial cell factories are presently upcoming [45]. Applications towards ethanol and biomass [46] and amino acids [47] were described, while sampling, as a critical topic has been recently highlighted [48]. Systems biology as the framework over all these global analytical approaches has been summarized in an editorial in 2007 [49].

Process modelling and design enable the transfer of cell factories towards production processes. Efficient substrate utilization is the first prerequisite for an efficient process [50]. While Cos et al. comprehensively reviewed process operation, monitoring and control for recombinant yeasts [51], modelling has been applied to optimize fed batch processes [52]. Advanced monitoring [53] will enable novel approaches for process optimization and control, while miniaturization of bioreactors [54] bears the chance of a wide expansion of experimental screening and design.

Many important reports published in the journal concerned molecular biology and genetics, including development of biosensors. In the field of analysis and modification of nucleic acids, improved methods for quantitative analysis of yeast RNAs [55], monitoring of gene expression in *Pichia pastoris *[40], determination of plasmid copy number [56] and generation of site-directed point mutations in *Escherichia coli *chromosome [57] were developed. Genomic and metabolomic procedures became more and more important in molecular biology and biotechnology. Topics concerning genomics of as diverse organisms as humans [58] and *Propionibacterium freudenreichii *[59] were addressed. Moreover, effects of the presence of plasmid DNA on metabolism of *E. coli *were reported [60].

Several excellent papers published in the journal concerned microbial gene expression. These include reports on regulation of expression of heat shock genes [2] and the *desA *gene [61]. A group of very interesting reports may be of high practical importance in the biotechnological use of microbial cell factories. Namely, it was shown that decreased recombinant gene expression from T7 promoters may be due to impaired production of active T7 RNA polymerase in the expression system [62], and that a transient increase of ATP level occurs in response to a temperature up-shift in *E. coli *[63]. Moreover, several new cloning vectors and expression systems were developed and described (see, for example references [11,64-67]). Finally, novel methods of sample analysis are especially important in the field of current applied microbiology. This was reflected by appearance of several articles in the journal, which were devoted to studies based on various biosensors and can be exemplified by electric bio-chips [68].

Apart from original reports, mentioned above, a group of review articles was published, in which current problems of molecular biology and genetics were discussed in the light of the microbial cell factory concept. Some of them touched the concept directly [69-71], while other were devoted to discuss cell factories in the light of stress responses [72,73], protein modification and quality control [4,6], regulation of gene expression [74,75], metagenomics [76], development and production of recombinant vaccines [77], and modern biosensors and analytical methods [53,78].

On the other hand, a large number of relevant reports have dealt with to the use of microorganisms for the production of natural molecules. Various reviews were devoted to microbial polysaccharides, whose importance in biotechnology is rapidly increasing: among these, one excellent paper by Ruffing and Chen [79] reported on numerous efforts made to enhance oligo- and poly-saccharide production in different microorganisms. Various articles were also devoted to the synthesis of amino acids of relevant industrial interest: over-production of phenylalanine, glutamate and most recently lysine [47] was studied in engineered *E. coli *strains and in *Corynebacterium glutamicum*. Various authors reported on the over-production of molecules of interest for food and human health by organisms such as *Escherichia *and *Proteus *and most recently Marx et al. [65] reported on the accumulation of riboflavin by a genetically engineered strain of *Pichia pastoris*. A very interesting review of works focusing on the use of various yeasts as cell factories for the production of fine chemicals and active pharmaceutical ingredients was published very recently by Pscheidt and Glieder [80]. *Saccharomyces cerevisiae *was also used by Branduardi et al [34] to study lactate production. In 2005, a review by Dutta et al [81] focused on hydrogen production by cyanobacteria, another field of high biotechnological interest in these times of energy shortage. Finally a new very stable bacteriocin of particular interest as an antimicrobial agent for solid foods and for industrial operation at high temperature was described by Martirani et al [12].

During these years Microbial Cell Factories has given a significant contribution also to the description of new or improved microbial strains for biotechnological applications. Sivaprakasan et al [82] reported on the use of a consortium of salt-tolerant microorganism for saline wastewater treatment. Among others, relevant impact had the construction of engineered strains of *Streptomyces pilosus *[61], *Lactococcus lactis *[64] and *Escherichia coli *[28,57,67] for the control of gene expression aimed at enhancing industrial productions. Optimization of industrial media for yeast and *Bacillus *was reported by Hahn-Hagerdal et al [50] and Zweers et al [14], respectively.

Regarding food microbiology, the journal has published research articles on food-related microbes' metabolism and physiology issues of industrial importance. In this context, a poorly characterized pathway for sucrose utilization in *Saccharomyces cerevisiae *was engineered to improve biomass-directed applications of the organism, in the work of Badotti et al [29]. Modulation at the molecular level of the rate of active sucrose uptake resulted in yeast strains that can easily attain higher cell densities with elevated sucrose levels avoiding overflow metabolism. The regulatory features of the pathways involved in methanol utilization by yeasts have been extensively reviewed by Hartner and Glieder [83]. The applications of methylotrophic yeasts are expanding today, beyond the established for single cell protein and recombinant protein production, to the biopharmaceuticals area and the production of therapeutic antibodies. The key role of thioredoxin reductase in the oxidative stress response of *Lactobacillus plantarum *was reported by Serrano et al [84]. The authors proposed that overproduction of the *trxB1*-encoded TR in *Lactobacillus plantarum *improves tolerance towards oxidative stress. This latter property can be used for engineering robustness towards oxidative stress in industrial strains of *L. plantarum*. Also, the adaptation to heat shock in *L. plantarum *was studied [85].

On the other hand, the identification or microbial metabolites produced during the food fermentation or during bioprocesses can be highly important regarding food additives or potential nutraceuticals. A genomic search approach that combined methods based on automatic and manual searches of homology and motifs among *Propionibacterium freudenreichii *was presented [59] as a tool for the prediction-identification of esterases, the lypolytic enzymes involved in Emmental cheese maturation and flavour development. Vanillin production and optimization using metabolically engineered *E. coli *have been also reported [86]. Ferulic acid was efficiently converted to vanillin in the reported work, without accumulation of undesirable vanillin reduction/oxidation products, using *E. coli *JM109 cells expressing genes from the ferulic acid-degrader *Pseudomonas fluorescens *BF13. Selection for spontaneous roseoflavin-resistant mutants was found to be a reliable method to obtain natural riboflavin-overproducing strains of a number of species (*Lactobacillus *(*Lb*.) *plantarum*, *Leuconostoc *(*Lc*.) *mesenteroides *and *Propionibacterium *(*P*.) *freudenreichii*) commonly used in the food industry [87]. The use of such starter strains can be exploited to increase the vitamin content in certain food products. Microbial exopolysaccharides make an important class of food components. They are produced by dairy (*Lactococcus *and *Streptococcus *spp.) and non-dairy bacteria, and in-situ synthesis allows modulation of rheology, improved mouthfeel, and texture of food products while it imparts some health benefits as prebiotics. Metabolic engineering strategies and various production aspects have been extensively reviewed [79]. Other classes of natural products that are increasingly becoming the centre of attention of the pharmaceutical and nutraceutical industries are isoprenoids, flavonoids and long chain polyunsaturated fatty acids. The use of *S. cerevisiae *as a cell factory for the biosynthesis of these high-value natural products has been also reviewed in Microbial Cell Factories [88].

The microbial production of enzymes used in the food industry has also had a place in the journal. Secretion and properties of a hybrid *Kluyveromyces lactis*-*Aspergillus niger *beta-galactosidase has been reported [89], as well as various aspects of the industrial scale production of chymosin by *Aspergillus niger *[90]. Overproduction of the important for the food industry pectin lyase was achieved by encapsulation of recombinant strains of *E. coli *in alginate or alginate/silica beads [91]. Regarding the exploitation of lactic acid bacteria in the production of valuable biotechnological products, contributions have reported the optimization of the nisin controlled gene expression system NICE of *Lactococcus lactis *for industrial purposes [64] and the applications of this system, ranging from membrane proteins to large scale processes [92], were also described.

Regarding food safety, a method based on electrochemical detection on a biochip enabling a fast characterization and monitoring of pathogens (microbial cells and spores) in food has been reported [68], as well as the evaluation and comparisons of bioassays and indicator microorganisms used in bacteriocin determination in processed food samples [93].

In summary, Microbial Cell Factories is offering a new open forum for the presentation and discussion of novel tools and scientific concepts regarding the use of microorganims for the production and transformation of biotechnologically relevant substances. The role of the journal, supported by an international, well experienced editorial board will keep being the publication of manuscripts of relevant and general interest in the Cell Factory context but also the cooperation with scientific conferences and the promotion of any way of scientific communication in this and related fields. We highly appreciate the valuable support of the authors, readers, referees, editors and the whole scientific community in these early stages of the journal development and consolidation.

## Competing interests

The authors declare no competing interests.
